# OM‐85, a Bacterial Lysate, Reduces Pulmonary Nodule Malignant Probability: A Retrospective Study

**DOI:** 10.1111/crj.70109

**Published:** 2025-07-21

**Authors:** Mengting Sun, Yuqing Ni, Xueling Wu, Hao Tian, Yijun Song, Yinzhou Feng, Yunxin Guo, Yong Zhang, Jun Yin, Charles A. Powell, Chunxue Bai, Yuanlin Song, Dawei Yang

**Affiliations:** ^1^ Department of Pulmonary and Critical Care Medicine Zhongshan Hospital Fudan University Shanghai China; ^2^ Department of Pulmonology, Renji Hospital Shanghai Jiaotong University School of Medicine Shanghai China; ^3^ School of Information Science and Technology Fudan University Shanghai China; ^4^ Department of Thoracic Surgery Zhongshan Hospital Fudan University Shanghai China; ^5^ Pulmonary, Critical Care and Sleep Medicine Icahn School of Medicine at Mount Sinai New York New York USA; ^6^ Shanghai Institute of Respiratory Diseases Shanghai China; ^7^ Department of Pulmonary and Critical Care Medicine, Zhongshan Hospital (Xiamen) Fudan University Xiamen Fujian Province China; ^8^ Department of Medical Internet of Things (MIOT) of Shanghai Respiratory Research Institution Shanghai China; ^9^ Shanghai Key Laboratory of Pulmonary Inflammation and Injury Shanghai China; ^10^ Shanghai Institute of Major Infectious Diseases and Biosafety Shanghai China; ^11^ National Clinical Medical Research Center for Geriatric Diseases Shanghai China; ^12^ Department of Pulmonary and Critical Care Medicine Jinshan Hospital Fudan University Shanghai China; ^13^ Shanghai Engineering Research for AI Technology for Cardiopulmonary Diseases Shanghai China

**Keywords:** immunomodulator, lung nodules, malignant progression, OM‐85

## Abstract

**Introduction:**

The current clinical management of pulmonary nodules relies heavily on CT follow‐up, without early intervention. This retrospective study investigated the efficacy of OM‐85, a standardized lysate of human respiratory bacteria, in the treatment of high‐risk pulmonary nodules detected by computed tomography (CT) in patients with chronic bronchitis.

**Methods:**

This study included 72 patients (93 enrolled nodules) who underwent treatment with OM‐85 and a matched control group of 90 patients (111 control nodules). The primary endpoint included reduced size of high‐risk ground glass nodules based on thin‐layer CT scans during follow‐up. Flow cytometry, multiplex immunofluorescence (mIF) analysis, and scRNA‐seq data were employed to determine differences in the immune cell subsets between the treatment and control groups.

**Results:**

Oral OM‐85 treatment significantly reduced lung nodule diameter (*p* = 0.031), the risk probability of malignancy (*p* = 0.003), and the likelihood of clinical disease progression (*p* = 0.0091). The effects of OM‐85 treatment were more pronounced in older patients (> 65‐year‐old) (*p* = 0.029) and those with longer follow‐up cycles (> 200 days) (*p* = 0.011). The peripheral blood samples showed a significantly higher proportion of natural killer (NK) cells in the treatment group. Furthermore, mIF staining of the pulmonary nodules and scRNA‐seq data demonstrated a higher percentage of NK cells in the treatment group compared with the control group (*p* = 0.0003).

**Conclusion:**

OM‐85 reduced the size of high‐risk pulmonary nodules and decreased the risk of malignant probability and disease progression in patients with chronic bronchitis by increasing the proportion of NK cells. Therefore, OM‐85 is a potential drug for the treatment of high‐risk pulmonary nodules in patients with chronic bronchitis.

## Graphical abstract

OM‐85 reduced the size of high‐risk pulmonary nodules and decreased the risk of malignant probability and disease progression by increasing the proportion of NK cells. Therefore, OM‐85 may be a potential agent for the treatment of high‐risk pulmonary nodules.

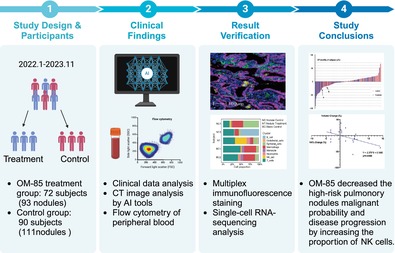

## 1 Introduction

Low‐dose computed tomography (LDCT) is a highly effective diagnostic tool for the early detection of lung cancer and can reduce the mortality rate by up to 20% [1]. In the National Lung Screening Trial (NLST), more than 53 000 people considered at high risk of lung cancer were screened with LDCT at 33 centers in the USA. Over three annual screenings, noncalcified lung nodules, measuring at least 4 mm, were detected in 24.2% of the study population, and approximately 1% of these nodules were diagnosed as malignant [2]. The clinical use of LDCT has coincided with an increasing prevalence of lung cancer cases worldwide [3].

Since pulmonary nodules can progress to early adenocarcinoma, clinical evaluation and management of incidental pulmonary nodules is critical [4]. Several risk probability models have been standardized for the regular screening of pulmonary nodules and the early diagnosis of lung cancer [5]. In this study, high‐risk lung nodules were defined as nodules with a high probability of malignancy evaluated by AI tools. The current paradigm for treating pulmonary nodules involves regular follow‐up and surgical resection, but this approach is associated with a high risk of overdiagnosis and increased radiation exposure [6]. Moreover, this approach causes significant psychological pressure on patients. Therefore, there is an urgent need to develop effective early intervention strategies for the clinical management of high‐risk pulmonary nodules.

OM‐85 (Broncho‐Vaxom) is a commercially available over‐the‐counter oral preparation generated by lysing multiple common respiratory bacterial pathogens. It is mainly used in the prevention and treatment of chronic airway diseases such as recurrent respiratory tract infection, asthma, and chronic bronchitis. It triggers both innate and adaptive immune responses and enhances the activity of peripheral blood monocytes and alveolar macrophages [7]. Previous studies have shown that oral OM‐85 administration promotes the maturation of T lymphocytes, restores the normal T lymphocyte helper/suppressor ratio, and improves T lymphocyte reactivity [8]. OM‐85 also enhances natural killer (NK) cell activity in peripheral blood [9].

NK cells are an important component of the innate immune system [10]. NK cells can contribute directly to immunity by autonomously targeting foreign, infected, or cancerous cells and releasing cytotoxic particles that directly trigger cell lysis, as well as indirectly influencing the immune response through the production of inflammatory cytokines, such as interferon gamma (IFN‐γ) and tumor necrosis factor alpha (TNF‐α) [11].

Based on clinical observations during immune‐stimulation therapy of patients with chronic bronchitis, we performed a retrospective population‐based study, using an artificial intelligence (AI) CT imaging analysis algorithm, to determine whether treatment with OM‐85 decreased the volume and/or diameter of high‐risk lung nodules and reduced the risk probability of lung cancer.

Although the immunomodulatory effects of OM‐85 have been established in the management of chronic respiratory infections and allergic asthma, its impact on pulmonary nodules and the immune microenvironment remains unexplored. This study is to specifically investigate the therapeutic potential of OM‐85 in chronic bronchitis patients with high‐risk pulmonary nodules and to elucidate its potential mechanisms for modulating the pulmonary immune microenvironment. Furthermore, the findings hold promise for offering a novel immunomodulatory strategy for patients at high risk of pulmonary nodule progression.

## 2 Methods

### 2.1 Study Design and Participants

**FIGURE 1:**
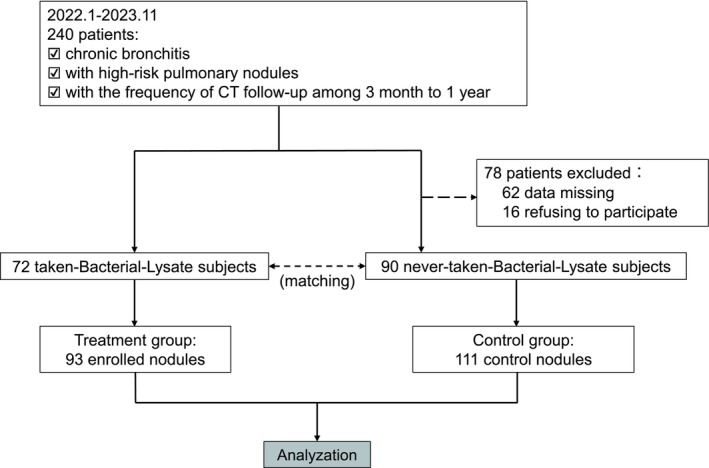
Flowchart of subject enrollment and screening.

This single‐center retrospective clinical study was conducted at the Zhongshan Hospital Fudan University, Shanghai, China. We analyzed the changes in the imaging, histological, and laboratory data of a cohort of chronic bronchitis patients with concomitant pulmonary nodules before and after adjunctive treatment with oral OM‐85. We investigated the therapeutic effects on pulmonary nodules and the adverse reactions associated with oral OM‐85 administration in this patient population. The study group did not undergo any specific interventions during the clinical data collection process. The overall enrolment flowchart is shown in Figure 1.

### 2.2 Inclusion and Exclusion Criteria and Informed Consent

We consecutively included 240 chronic bronchitis patients who were treated at the Department of Respiratory Medicine from January 2022 to November 2023. The inclusion criteria were as follows: (1) patients diagnosed with chronic bronchitis by clinical physicians; (2) confirmatory diagnosis of high‐risk lung nodules evaluated by the AI tool based on thin‐layer CT scans (specifically, nodules with a diameter > 5 and ≤ 20 mm that were stably detected by radiologists for at least 3 months and did not show natural disappearance); and (3) availability of thin‐layer CT follow‐up records, especially thin‐layer CT scans conducted at our hospital with a follow‐up period of between 4 and 12 months. The exclusion criteria were as follows: (1) target lung nodules with clear benign morphological features, including uniform calcification as well as solid nodules with regular and round or polygonal edges; (2) subjects diagnosed with malignant diseases either currently or within the past 5 years; (3) patients being treated concurrently with antibiotics during the OM‐85 intake; (4) patients with missing data or who refused to participate or those who were not reachable by telephone.

The potential participant list was retrieved from the medical records system and the CT reports were screened to identify the individuals with nodules displaying the required characteristics.

In the treatment group, patients were prescribed one oral capsule of OM‐85 daily for 10 days/month. Patients took the OM‐85 capsule for 10 consecutive days and then stopped for 20 days. This cycle was repeated monthly for 3 months. The control group patients did not receive any drug intervention. Each patient's adherence to the medication was confirmed by telephone follow‐up.

Patients with high‐risk lung nodules detected by thin‐layer CT scans underwent regular follow‐ups by thin‐layer CT at our hospital, with intervals ranging from 3 to 12 months. For subjects with more than one eligible target nodule on the thin‐layer CT images, all eligible nodules were included in the final analysis.

The follow‐up CT images and the original chest CT images at baseline were checked to ensure that they met the requirements of the AI diagnostic system and were devoid of significant respiratory motion artifacts.

Upon the occurrence of any adverse event, the case report form is duly completed with the relevant details. Subsequently, the aforementioned adverse events are incorporated into the monthly adverse event report form and the case report form.

All included patients signed the informed consent form or provided consent by telephonic conversation.

### 2.3 CT Image Acquisition

All included patients underwent unenhanced chest CT examination and a whole lung scan. The scans were obtained with deep inspiration breath hold. The CT images were obtained using one of the following four CT scanners: Brilliance 64 (Philips Medical Systems Inc., Netherlands), SOMATOM Definition AS (Siemens AG, Munich, Germany), SCENARIA (Hitachi Ltd., Tokyo, Japan), or Aquilion ONE (Canon Medical Supply Co. Ltd, Tokyo, Japan). The CT scan parameters for the above devices were as follows: tube voltage, 120–130 kV; tube current, 100–150 mAs; rotation time, 0.5–0.75 s; pitch, 0.828–1.2; matrix, 512*512 and standard resolution algorithms; reconstruction kernel of lung window: standard (B) for Brilliance 64, B60f sharp for SOMATOM Definition AS, Lung Sharp for SCENARIA, and Lung‐Std Axial for Aquilion ONE; lung window settings (width/level), 1200/−600 HU; mediastinal window settings (width/level), 400/40 HU; voxel dimensions, 1 × 1 × 1 mm.

### 2.4 Clinical Data Analysis

The clinical data of enrolled patients, including demographic characteristics (age, gender, smoking status, and alcohol consumption), medical history (duration of lung nodule discovery, history of respiratory illness, surgical history, COVID‐19 infection history, comorbidities, and the timing of hospital visits), biochemical indicators (the percentages of immune cells), and treatments (steroids, antibiotics, and inhaled medications), were retrieved from the electronic medical records. The present and past medication history was obtained by answering questionnaires through telephone follow‐up.

The baseline and follow‐up CT data of the patients were uploaded to an independent server for semiautomated AI image analysis to estimate the nodule diameter, density, distance from the pleura, and the lung cancer risk score (ranging from 0% to 100%). A diagnostic statement was also obtained for the target lung nodules based on these estimates.

### 2.5 CT Image Analysis

Imaging data for the enrolled subjects were uploaded to the Target‐Call Lung Nodule Analysis System (https://ctai.sanmedcloud.com), an “AI‐based lung nodule analysis system” developed by the Zhuhai Heng‐qin SanAo Cloud Wisdom Technology Co. Ltd. This system is based on deep learning AI algorithms and involves a three‐stage end‐to‐end deep conventional neural network (DCNN) to analyze the LDCT images of the patients. A previous study reported that combining AI risk scores with radiological features improved the early‐stage diagnosis of lung cancer with a sensitivity of > 80% [12]. The AI diagnostic software independently performed image analysis and malignancy diagnosis by automatically recognizing, labelling, and diagnosing lung nodules. Subsequently, the AI software was used to generate a risk score (ranging from 0% to 100%) and a diagnostic statement for the target lung nodules of each participant. Subsequently, statistical analyses of the AI‐generated lung nodule parameters were performed. Chi‐square analysis was performed to assess the differences in the follow‐up outcomes for the target lung nodules between the control and treatment groups.

### 2.6 Multiplex Immunofluorescence (mIF) Staining

To identify differences in the NK cells between the two groups of patients (two patients received OM‐85 oral therapy, and two patients had never received it) within the malignant pulmonary nodules environment, mIF staining was obtained using PANO 7‐plex IHC kit, cat 0004100100 (Panovue, Beijing, China). The slides were sequentially incubated with different primary antibodies (PanCK, Cell Signaling Technology, #4545; CD16, Cell Signaling Technology, #24326; CD56, Cell Signaling Technology, #3576). Subsequently, the samples were incubated with the horseradish peroxidase‐conjugated secondary antibody followed by tyramide signal amplification. The slides were microwave heat‐treated after each TSA operation. The nuclei were stained with 4′‐6′‐diamidino‐2‐phenylindole (DAPI, Thermo Scientific) after all the human antigens were labelled [13]. The stained slides were scanned using the Vectra System (PerkinElmer), and fluorescent signals were collected for all the samples between 420 to 720 nm. InForm image analysis software (PerkinElmer, Waltham, Massachusetts, USA) was used to analyze the data. GraphPad Prism 8 software was used to plot the data.

### 2.7 Single‐Cell RNA‐Sequencing Analysis

To determine the number of localized NK cells in the lung, single‐cell sequencing was performed on fresh human lung tissue specimens. Specifically, after obtaining informed consent, we obtained surgical specimens from patients with malignant lung nodules (including nodule tissue samples and normal lung tissue specimens 3 cm adjacent to the nodule). One patient received OM‐85 oral therapy, and two patients had never received it.

Single‐cell suspensions of samples (fresh high‐risk pulmonary nodules and normal tissues resected from patients) were resuspended in 1× PBS containing 0.04% BSA at 5 × 10^5^ cells/mL and barcoded with a 10× Chromium Controller (10× Genomics). The raw sequencing data was demultiplexed using the default settings in the 10× Genomics Cell Ranger workflow and mapped to the reference genome [14]. Cell Ranger and Seurat were used for all the downstream single‐cell analyses. The scRNA‐seq dataset combinatorial analysis was performed on five samples (bc1/bc2/NC1/NC2/NT) from the Chinese Academy of Sciences (GSA‐Human), reference number (HRA005794). For quality control, cells expressing at least 200 genes were selected. Moreover, only genes expressed in ≥ 3 cells were considered. Low‐quality cells were first filtered using the following threshold parameters: nFeature_RNA > 300 and < 7000; nCount_RNA < 100 000; mitochondrial content < 10% and red blood cell content < 3%. Batch effects were corrected using the R4.0.5 packet Harmony. Subsequently, the identified clusters were visualized on the 2D map produced with the UMAP method. To annotate the cell clusters, we used the R package Single R for automatic annotation of cell types.

### 2.8 Statistical Analysis

In this study, patients with a history of supplementation with oral OM‐85 were assigned to the treatment group (*n* = 72), and those without supplementation with OM‐85 were assigned to the control group (*n* = 90). If more than one eligible target nodule was identified in a study subject based on thin‐layer CT results, all the eligible nodules were included for the analyses. The demographic characteristics and medical history of the two groups are shown in Table 1.

**TABLE 1:** Demographic and clinical characteristics of the study subjects.

Demographic or clinical characteristics of the study subjects	Control group	Treatment group	*P*‐value
	*n* = 90	*n* = 72	
**Gender, *n* (%)**			
Male	32 (35.6)	34 (47.2)	ns
Female	58 (64.4)	38 (52.8)	
**Age, y**			
Average	52.48 ± 15.27	51.96 ± 15.27	ns
Median	55.50	51.00	
Interquartile range	30	25	

The primary endpoint of this study was changes during the 6‐month follow‐up in the high‐risk lung nodules, which were detected in the baseline thin‐layer CT scans. The AI imaging analyses ensured stability and repeatability of the readings. The AI imaging algorithm was used to measure the volume and density of the lung nodules and estimate their lung cancer risk probability. Clinical remission based on nodule reduction was evaluated using the Response Evaluation Criteria In Solid Tumors (RECIST) criteria [15]. As these criteria apply to tumors, we applied them selectively for the clinical features of malignant lung nodules (Table S1A,B). Disease control rate (DCR) was defined as the percentage of cases in which remission (PR + CR) and stability (SD) of lesions were achieved after treatment. Descriptive statistics for the categorical data were analyzed using the contingency table with the Fisher's exact test. The 95% confidence intervals (95% CIs) for the proportions were estimated using the exact binomial method. Nonparametric tests were used to compare the ordered data with trends. Continuous data were analyzed using the student's *t*‐test. The Shapiro–Wilk test was used for normality testing of the continuous variables. *p* < 0.05 was considered statistically significant.

The RECIST criteria state that to evaluate the efficacy of a tumor treatment, easily measurable target lesions should be selected and the sum of the longest diameters (SLD) of each lesion should be calculated. At posttreatment imaging, the change in SLD value of the target lesion is compared to determine the presence or absence of nontarget lesions and to look for new lesions. Efficacy is then determined: complete remission (CR), partial remission (PR), stable disease (SD), and disease progression (PD).

According to the criteria above, the presence of any new and well‐defined malignant tumor lesion indicates disease progression. If the new lesion is ill‐defined, for example, due to small size, further evaluation will clarify the cause. If the evaluation of a definite lesion is repeated, then progression should be documented on the date of the first evaluation. Lesions found in previously unscanned areas were considered new lesions. Since this study was mainly aimed at the evaluation of isolated lung nodules, without considering tumor dissemination and implantation or evaluating lymph node metastasis, the evaluation criteria were simplified by simply measuring the longest diameter of the target lung nodule and taking the results of the two thin‐layer CT follow‐up images as the evaluation time points. The simplified evaluation criteria are summarized below.

## 3 Results

### 3.1 Baseline Characteristics of the Included Study Subjects

This study initially enrolled 1036 patients with chronic bronchitis and concomitant lung nodules from the Respiratory Medicine Department of the Zhongshan Hospital, Fudan University, between January 2022 and November 2023. Among these, 240 participants met the criteria for the nodule characteristics and underwent follow‐up CT scans at the required frequency. Among these, 62 patients were excluded because of missing thin‐layer CT data in the database, and 16 patients declined to participate. Finally, over a 14‐month period, data for 93 lung nodules from 72 eligible participants were included in the treatment group. These participants showed a history of oral OM‐85 intake as part of the adjunctive treatment based on the hospital medical record system and reviewed by the telephone follow‐ups. Subsequently, based on the baseline data of the treatment group, 90 patients who adhered to the prescribed follow‐up management regularly in accordance with the current clinical guidelines but did not take oral OM‐85 were selected for the control group from the medical records system. The flowchart of patient selection is shown in Figure 1.

The baseline demographic and clinical characteristics of the included study participants are shown in Table 1A, and baseline clinical features of nodules are shown in Table 1B. The treatment and control groups did not show statistically significant differences in parameters such as median age, gender distribution, smoking history, cancer history, clinical symptoms, baseline nodule size, nodule location, and risk probability (all *p* > 0.05).

**TABLE 1:** Baseline clinical features of nodules.

Baseline clinical features of nodules	Control group	Treatment group	*P*‐value
	*n* = 111 (from 90 patients)	*n* = 93 (from 72 patients)	
**CT surveillance time, y**			ns
Average	3.08 ± 2.74	2.82 ± 2.48	
Median	2.00	2.00	
Interquartile range	3.0	3.0	
Nodule diameter*, mm			
Mean	7.72 ± 2.66	7.52 ± 2.75	ns
Median	7.30	7.00	
Interquartile range	2.9	2.8	
**Nodule location** ^**a**^ **, number (%)**			
LLL	35 (31.5)	26 (28.0)	ns
LUL	15 (13.5)	10 (10.8)	
RLL	15 (13.5)	17 (18.3)	
RML	9 (8.1)	7 (7.5)	
RUL	37 (33.3)	33 (35.5)	
**Baseline probability of malignancy** ^**b**^ **, %**			
Mean	70.62 ± 16.54	72.03 ± 15.47	ns
Median	70.00	70.00	
Interquartile range	24.00	28.00	
**Follow‐up period, months**			
Mean	7.213 ± 3.28	6.844 ± 0.11	ns
Median	6.43	5.90	
Interquartile range	5.83 to 8.64	5.06 to 8.40	

^a^ LLL, left lower lobe; LUL, left upper lobe; RLL, right lower lobe; RML, right middle lobe; RUL, right upper lobe.

^b^ AI analyzed the images independently.

### 3.2 Treatment Group Showed Significant Reduction in the Malignant Risk Probability of the Lung Nodules

**FIGURE 2:**
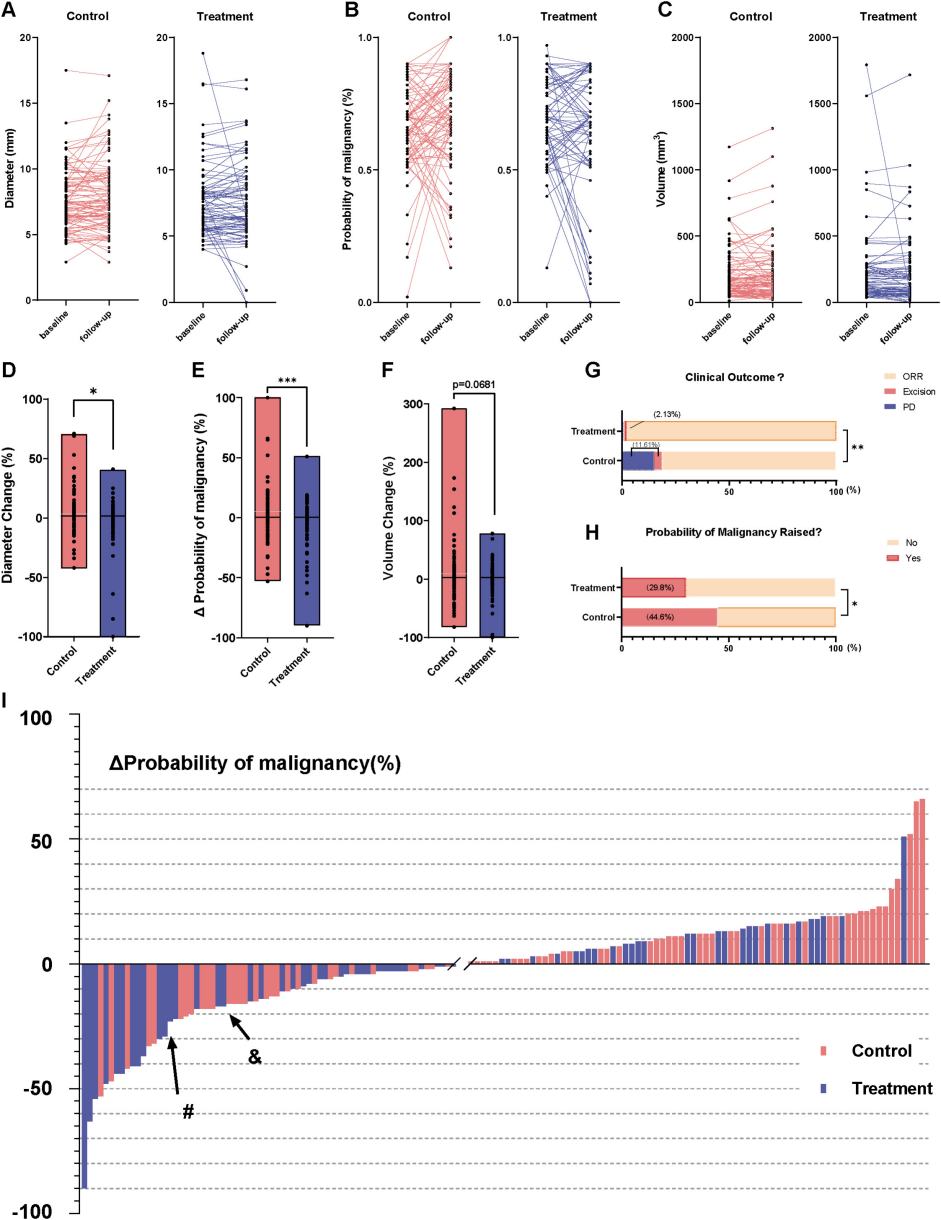
Differential changes in the characteristics of the target lung nodules in the control and treatment groups of patients at baseline and follow‐up (Control *n* = 111; Treatment *n* = 93). (A–C) Paired line plots of individual changes in (A) nodule diameter, (B) malignancy probability, and (C) volume (baseline vs. follow‐up). (D) Change in pulmonary nodule diameter. (E) Change in probability of pulmonary nodule malignancy risk. (F) Change in pulmonary nodule volume. (G) Difference in probability of increased malignancy risk for high‐risk pulmonary nodules between control and treatment groups. (H) Difference in clinical progression of high‐risk pulmonary nodules between control and treatment groups. (I) Waterfall plot of changes in probability of malignancy risk for high‐risk pulmonary nodules. The black arrows mark the position in the waterfall plot of the two patients mentioned in Figure 4.

The AI CT imaging data analysis demonstrated that the malignant risk probability of the lung nodules was significantly reduced in the treatment group compared with the control group. Target nodules in the treatment group exhibited a more pronounced reduction trend during follow‐up, as visually depicted in the paired line plot (Figure 2A) and further supported by the between‐group comparison in the boxplot (Figure 2D, *p* = 0.031). Furthermore, comprehensive analysis using the AI‐learned risk probability model showed a significant reduction in the malignant risk probability of the lung nodules in the treatment group compared with the control group (*p* = 0.003) (Figure 2B,E). At the same time, the volume change of lung nodules in the treatment group also showed a downward trend (*p* = 0.0681) (Figure 2C,F). Subsequently, the revised RECIST criteria were used to evaluate the treatment effects. Compared with the control group, the treatment group showed a higher likelihood of a significant reduction in the risk probability of the lung nodules (*p* = 0.0286) (Figure 2G). Specifically, only 30.1% (28/93) of cases in the treatment group demonstrated increased risk probability and clinical progression of lung nodules, whereas 45.0% (50/111) of cases in the control group showed increased malignancy risk probability (OR = 1.901, 95% CI: 1.051 to 3.424). Furthermore, the probability of clinical disease progression was significantly lower in the treatment group compared with the control group (*p* = 0.0091; Figure 2H; Table S2). Overall, the waterfall plot showed that the treatment group was more likely to reduce the probability of malignant lung nodules compared with the control group (Figure 2I).

### 3.3 OM‐85 Significantly Increased the Proportion of NK Cells in the Peripheral Blood and the Resected Lung Nodule Samples From Patients

**FIGURE 3:**
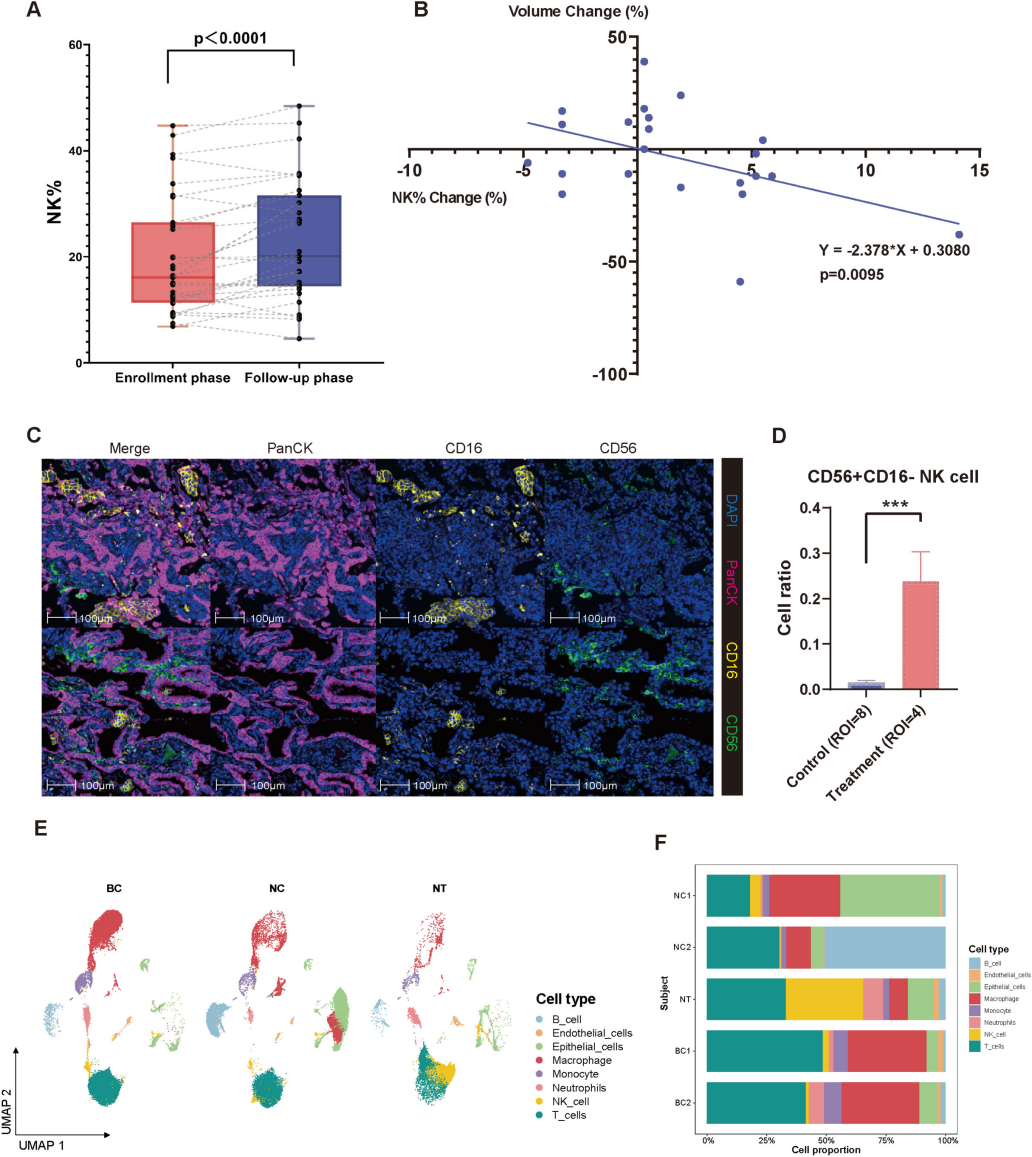
Oral OM‐85 treatment induces significant changes in the immune cell subsets during follow‐up of the treatment group patients. (A) The percentages of the NK cell subsets in the peripheral blood of patients in the treatment group at baseline and follow‐up. The y‐axis represents percentage of NK cells; the x‐axis displays different immune cell subsets; the enrollment phase and follow‐up data are represented with distinct colors. A combination of box plot and scatter plot shows the percentage of NK cells at baseline and follow‐up. The dashed lines connect data points from the same patient (*n* = 33). (B) The relationship between the changes in the percentage of NK cells (x‐axis) and changes in the nodule volumes (y‐axis) of patients in the treatment group. (C) Representative mIF images (20× magnification) showing differences in the infiltration of NK cells in the pulmonary nodule region between the control and treatment groups. Note: blue, DAPI; pink, PanCK; yellow, CD16; and green, CD56. The control groups included blank control (ROI = 4) and antibiotic control (ROI = 4). (D) The histograms showing differences in the proportion of CD56+ NK cells in the intratumoral regions of pulmonary nodule samples between the control group (ROI = 8) and the treatment group (ROI = 4). The data in 3D represents the quantitative analysis of mIF data that is shown in (C) (*p* < 0.001). (E) Single‐cell sequencing data analysis showing the proportion of several types of immune cells. UMAP demonstrating the eight major cell types. (F) Percentage bar graph outlines the proportion of several types of immune cells in the normal lung tissues, pulmonary nodule areas of the untreated patient, and in the pulmonary nodule area of the treated patient. *p < 0.05; **p < 0.01; ***p < 0.001; ****p < 0.0001.

Previous studies have reported that the bacterial lysis products stimulated the immune cell subsets in the peripheral blood [16]. Therefore, we further investigated the potential mechanisms by which OM‐85 oral administration decreased the size of pulmonary nodules and reduced the risk probability in the chronic bronchitis patients. Patient‐specific information is shown in Table S3. Figure 3 shows the comparative changes in the immune cell subsets in patients undergoing OM‐85 therapy at enrollment and at follow‐up. Blood analysis data for 33 patients in the treatment group were retrieved, and a paired analysis was performed of changes in the peripheral blood lymphocyte subsets between the baseline and follow‐up periods (Figure S1A, B). Our data showed a significant increase from baseline in the percentage of NK cells during the follow‐up period after OM‐85 therapy (*p* = 0.0003) (Figure 3A).

A linear fit was made between the change in NK cell percentage and the change in nodule volume for patients in the treatment group. The increase in NK cell percentage correlated with the decrease in volume of the malignant lung nodule of interest (*p* = 0.0095) (Figure 3B).

Following assessment by thoracic surgeons, we obtained surgical specimens from four clinical patients (two in the control group and two in the treatment group) who were all pathologically diagnosed as stage IA lung adenocarcinoma. Subsequently, the tumor specimens were subjected to mIF staining analysis. The tumor regions were delineated by specialized pathologists (Figure 3C). The mIF staining results demonstrated that the number of CD56^+^ NK cells significantly increased within the tumor microenvironment in the treatment group, but there were no significant differences in the number of CD16^+^ NK cells (Figure 3D *p* < 0.001; Figure S1C).

Single‐cell sequencing analysis was performed with the nodule (NC1/NC2) and paranodule (bc1/bc2) samples from patients with untreated high‐risk pulmonary nodules and nodule samples from treated patients (NT). Our data showed significant changes in the proportion of different populations of immune cells at the transcriptome level in patients treated with OM‐85, and a significant increase in the number of NK cells (Figure 3E,F).

### 3.4 Progressive Reduction of the Lung Nodule Size and Risk Probability Based on CT Imaging Analysis of Patients Receiving Oral OM‐85 Treatment During Follow‐Up

**FIGURE 4:**
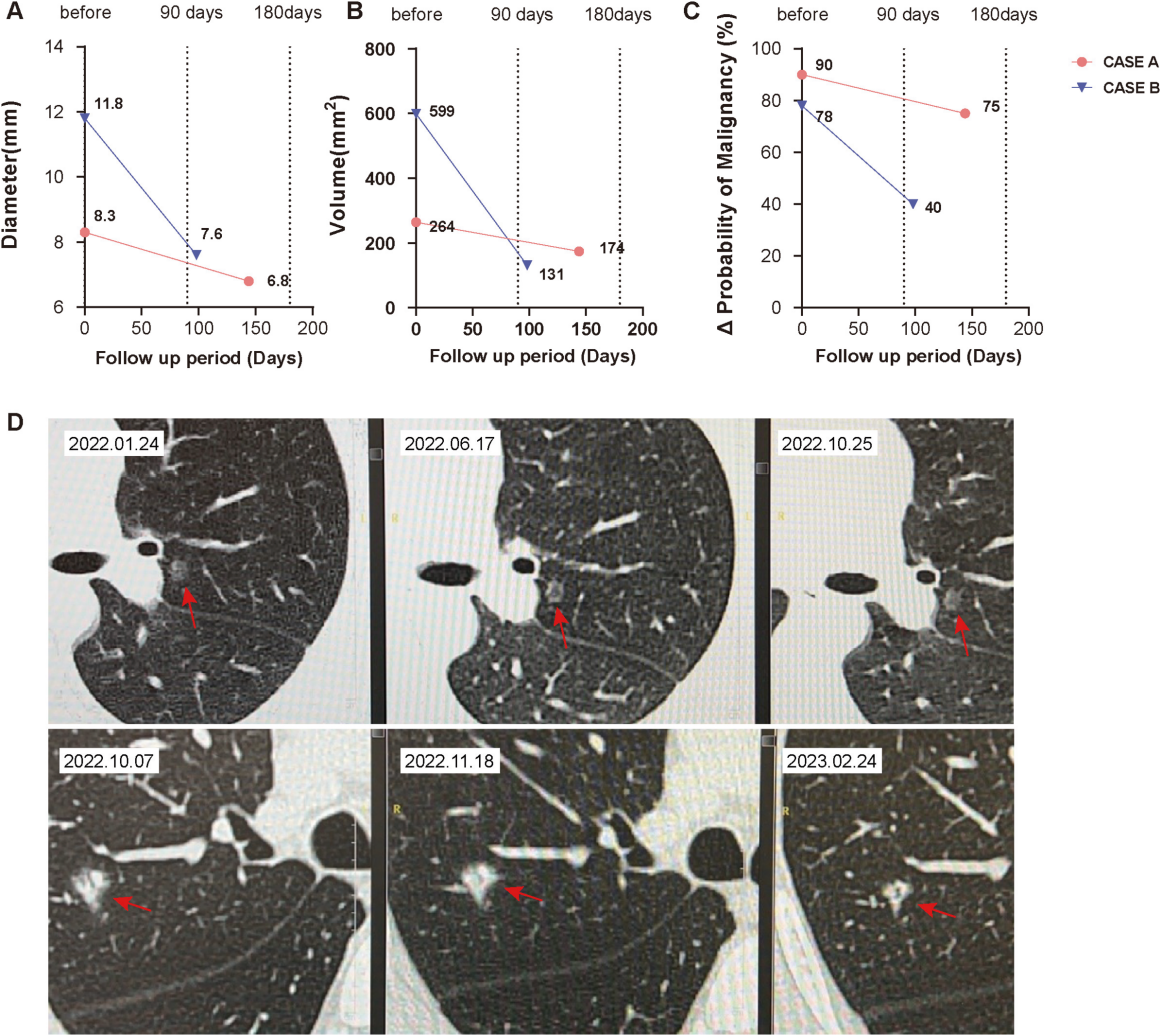
Clinical Improvement in the representative treatment group cases with chronic bronchitis and lung nodules. (A–C) The lung nodule diameter and the malignancy risk probability of Cases A and B with chronic bronchitis and concomitant lung nodules after receiving treatment with oral OM‐85. Specifically, diameter of the lung nodules and the malignancy risk probability as assessed by the AI image analysis decreased at 3 and 6 month compared to baseline. The x‐axis represents the follow‐up days. The segments of the lines represent the start of oral OM‐85 treatment and two follow‐up observation points. Specific numerical values are indicated in the figures. (D) Representative images of the lungs showing the high‐risk nodules (indicated by the red arrows) in Cases A (above) and B (below) at baseline, 3‐month, and 6‐month follow‐up time points. Each horizontal column represents one patient.

Figure 4 demonstrates changes in the diameter of lung nodules (labelled with a red arrow) and the risk probability of malignancy in two patients with chronic bronchitis who underwent treatment with OM‐85. Case A represents a mixed nodule, whereas Case B represents a solid nodule labelled with arrows (Figure 2F). To our knowledge, this is the first report to describe multiple cases of reduced lung nodule growth after treatment with an oral immunostimulant.

### 3.5 Follow‐Up Time and Age May Be Related to OM‐85 Treatment of High‐Risk Pulmonary Nodules

To further identify the factors that influence the clinical outcomes of the pulmonary nodules in patients treated with OM‐85, we performed a stratified analysis of the follow‐up outcomes for the target pulmonary nodules in both the control and treatment groups. A follow‐up period of 200 days was used as the cutoff between the long and short durations. To identify patients with a high risk of lung cancer, stratified analyses based on age were performed, and the study subjects were categorized as the younger (≤ 65 years) or older (> 65 years) age groups, using 65 years of age as the cutoff. The logistic regression stratified analysis was based on the interval between patient visits for the follow‐up CT scans (i.e., the follow‐up period) and age. Our data showed that a prolonged follow‐up period of > 200 days was significantly associated with decreased probability of malignant risk among the elderly patients (*p* = 0.011, *p* = 0.029) (Table 2).

**TABLE 2:** Logistic regression stratified analysis of follow‐up outcomes for the target pulmonary nodules in the control and treatment groups.

	Risk reduction	Control	Treatment	*P*‐value
**Follow‐up period** ≤ 200 days	Yes	41	37	0.694
	No	21	16	
**Follow‐up period** > 200 days	Yes	21	29	**0.011** *
	No	29	12	
**Age** ≤ 65 years	Yes	32	23	0.309
	No	45	47	
**Age** > 65 years	Yes	18	5	**0.029** *
	No	17	19	

* *p* < 0.05.

### 3.6 Safety

Of the 162 patients who were initially screened, two patients in the treatment group reported diarrhea, which was considered to be related to the study medication. This resolved after they ceased taking the medication. Subsequently, these patients were excluded from the final analyses due to the absence of data pertaining to the follow‐up period (Table S4).

## 4 Discussion

Air pollution exposure is a significant risk factor for chronic respiratory diseases and lung cancer. Currently, as air pollution becomes a global public health problem [17], the treatment of lung nodules is of paramount importance, but standardized guidelines for the diagnosis and treatment of lung nodules are currently lacking [18]. Early intervention methods for lung nodules are not listed in the National Comprehensive Cancer Network (NCCN) guidelines, and the classical recommendations are limited to regular thin‐layer CT follow‐up and surgical resection [19].

Therapy with immunostimulants has shown promise in significantly reducing the exacerbation of chronic bronchitis and chronic obstructive pulmonary disease, thereby reducing the need for antibiotic treatment [20]. OM‐85 is an immunostimulant that is primarily used for chronic bronchitis, wheezing episodes, and severe lower respiratory tract diseases [21]. We therefore retrospectively looked at high‐risk nodules in the lungs of patients who had been treated with OM‐85 for chronic bronchitis.

In our research, CT imaging analysis showed that patients with chronic bronchitis and lung nodules treated with the OM‐85 exhibited significant changes in lung nodules on CT imaging [22]. Furthermore, patients in the treatment group showed significantly higher proportions of NK cells in their blood, compared with the control group. NK cells are fundamental cellular mediators of anti‐tumor immunity [23]. Based on the hypothesis that OM‐85 kills aberrant cells by regulating the immune microenvironment [24], we focused on the dynamic interactions between cells and the immune microenvironment. We obtained postresection clinical samples and analyzed them by scRNA‐seq and mIF. In combination with a comprehensive atlas of the cellular composition of early spectrum lung cancer cells, we found an upregulation of the CD56+ subpopulation of NK cells. mIF staining confirmed this observation. This suggested that the OM‐85 reduced the size and malignancy probability of the lung nodules by enhancing the proportion and activity of the NK cells, thereby providing a new perspective for clinical intervention in chronic bronchitis patients with lung nodules.

In this retrospective study, we observed significant diagnostic heterogeneity between the enrolled subjects. Therefore, the control group selection was designed to eliminate as many confounding factors as possible [25]. To ensure that the retrospective study did not include spontaneously resolving inflammatory lesions, we selected persistent nodules based on continuous CT scans that were performed for two consecutive years [26]. Furthermore, we chose lung nodules with a diameter > 5 and ≤ 20 mm. These nodules were stable for over 3 months and did not show natural disappearance. We excluded nodules that had benign morphological features, such as uniform calcification and/or solid nodules with regular and round or polygonal edges. We also excluded subjects with current malignant diseases, those who had a malignant disease in the past 5 years, and patients who had been treated with antibiotics during the oral OM‐85 period.

This study also investigated the feasibility and interpretability of consecutive follow‐ups for the CT‐detected lung nodules. We used the modified RECIST criteria for very small lesions < 1 cm to classify the nodule response rates and assess the effectiveness of the intervention. Since the resected nodules represent suspicious lesions, all the resection events were defined as cases of clinical progression in our study. Furthermore, we did not subject patients to additional invasive procedures but performed multiple immunofluorescence staining of the pathology specimens from the resected nodules to determine the status of immune cell infiltration. However, the limited number of pathological cases remains a significant limitation in the study design.

Our study has some limitations. First, we tried to avoid enrollment bias by using strict enrollment criteria to precisely match the treatment group with the control group. However, we were unable to prospectively observe the clinical response of patients and avoid the loss of outpatients during follow‐up. Therefore, the sample size was small. Second, we used AI image scoring to estimate the malignancy risk of the lung nodules [27]. This technology is gaining wider acceptance and can ensure result stability and minimize subjective bias [28]. In clinic, the manual assessment of nodule diameter to estimate growth is not accurate, especially for nodules with irregular edges [14]. In order to minimize bias, we included lung nodule volume parameters as one of the endpoint indicators and used the semiautomatic volume measurement method provided by the AI analysis platform to ensure a more accurate assessment of lung nodule size and growth.

Our data showed that the immunostimulatory effects of OM‐85 treatment provided an opportunity for delaying aggressive malignant tumor treatment and demonstrated that the existing clinical follow‐up can be improved. The malignant probability is significantly decreased for lung nodules that show reduced growth over time. This can extend the required follow‐up period, and the treatment can be more conservative. If lung nodules remain stable, the frequency of CT examinations can be reduced, thereby alleviating the cost burden on the patients and reducing their anxiety [28]. This also promotes effective utilization of the available medical resources and increases the efficiency of healthcare management. This innovative treatment approach provides a fresh perspective for the diagnosis and treatment of lung nodules.

In conclusion, our study demonstrated that the oral OM‐85 significantly reduced the risk of progression to malignancy of the high‐risk lung nodules that were diagnosed by thin‐layer CT. Therefore, oral OM‐85 is a potential drug for preventing lung adenocarcinoma. Long‐term follow‐up showed that high‐risk lung nodules in patients treated with oral OM‐85 exhibited reduced nodule growth and volume, and had decreased the probability of clinical progression. Furthermore, the treatment group patients showed a higher number of NK cells in the resected samples compared with the control group, demonstrating immune activation in the pulmonary nodules in response to treatment with OM‐85. Our data demonstrated that the oral OM‐85 intervention may be a crucial tool in managing patients with high‐risk nodules.

Further research is required to investigate the mechanisms of action and to provide novel perspectives and options for the diagnosis and treatment of lung nodules. Furthermore, prospective randomized controlled trials with larger sample sizes are required to validate our results.

## Author Contributions

Mengting Sun and Yuqing Ni wrote the main manuscripts. Xueling Wu, Hao Tian, Yijun Song, Yinzhou Feng, and Yunxin Guo performed the data extraction and statistical analysis. Yong Zhang, Hui Kong, Shaohua Lu, Jun Yin, Charles A. Powell, Chunxue Bai, Yuanlin Song, and Dawei Yang contributed to the conception and design of the study.

## Ethics Statement

This retrospective clinical trial was approved by the Ethics Committee of Zhongshan Hospital, Fudan University, China (Approval No. B2022‐471 (2)).

## Conflicts of Interest

The authors declare no conflicts of interest.

## Supporting information


**Table S1A.** Criteria RECIST 1.1 for Classification of outcomes for evaluating the effectiveness of tumor treatments.
**Table S1B.** A simplified version of the RECIST criteria for categorizing outcomes in evaluating the therapeutic effects of drugs for pulmonary nodules.
**Table S2.** Comparison of lung nodule parameters between treatment and control groups.
**Table S3.** Details of patients whose samples were used for multiple immunofluorescences.
**Table S4.** Attributable toxicities. Number of events (number of patients).
**Figure S1.** The bar graph shows the absolute counts (A) and percentages (B) of NK cell subpopulations and CD8 cell subpopulations in the peripheral blood of patients in the treatment group at baseline and follow‐up. The Y‐axis represents the number or percentage of cells; the X‐axis shows the different immune cell subpopulations; and the enrolment stage and follow‐up data are indicated by different colors. (C) The histograms show differences in the proportion of CD16 + CD56‐ NK cells in the intratumoral regions of pulmonary nodule samples from the control group (ROI = 8) and the treatment group (ROI = 4). The data represent the quantitative analysis of mIF data that is shown in Figure 4C.

## Data Availability

The major data that support the findings of this study are available in the Chinese Academy of Sciences database at https://ngdc.cncb.ac.cn/gsa‐human/, under the accession number HRA005794. These data were derived from the following resources available in the public domain: https://ngdc.cncb.ac.cn/search/specific?db=hra&q=HRA005794. Additional single‐cell RNA sequencing data that are not publicly available due to patient privacy or ethical restrictions can be obtained from the corresponding author upon reasonable request.
