# Supplementary material for: OM‐85, a Bacterial Lysate, Reduces Pulmonary Nodule Malignant Probability: A Retrospective Study
Source: Clin Respir J. 2025 Jul 21;19(7):e70109. doi: 10.1111/crj.70109 (PMC12280052; doi:10.1111/crj.70109)
Supplement: Supplementary file 1 — Table S1A. Criteria RECIST 1.1 for Classification of outcomes for evaluating the effectiveness of tumor treatments. Table S1B. A simplified version of the RECIST criteria for categorizing outcomes in evaluating the therapeutic effects of drugs for pulmonary nodules. Table S2. Comparison of lung nodule parameters between treatment and control groups. Table S3. Details of patients whose samples were used for multiple immunofluorescences. Table S4. Attributable toxicities. Number of events (number of patients). Figure S1. The bar graph shows the absolute counts (A) and percentages (B) of NK cell subpopulations and CD8 cell subpopulations in the peripheral blood of patients in the treatment group at baseline and follow‐up. The Y‐axis represents the number or percentage of cells; the X‐axis shows the different immune cell subpopulations; and the enrolment stage and follow‐up data are indicated by different colors. (C) The histograms show differences in the proportion of CD16 + CD56‐ NK cells in the intratumoral regions of pulmonary nodule samples from the control group (ROI = 8) and the treatment group (ROI = 4). The data represent the quantitative analysis of mIF data that is shown in Figure 4C. [file CRJ-19-e70109-s001.docx]

**Supplementary figures and tables**

**Table S1A** Criteria RECIST 1.1 for Classification of Outcomes for Evaluating the Effectiveness of Tumor Treatments.

| Classification | Definition |
| --- | --- |
| CR (Complete response) | Disappearance of all lesions and pathologic lymph nodes |
| PR (Partial response) | >30% decrease SLD  no new lesions  no progression of non-target lesions |
| SD (Stable disease) | no PR - no PD |
| PD (Progressive disease) | ≥20% increase SLD* compared to smallest SLD in study or progression of non-target lesions  or new lesions |

The RECIST criteria state that to evaluate the efficacy of a tumor treatment, easily measurable target lesions should be selected and the sum of the longest diameters (SLD) of each lesion should be calculated. At post-treatment imaging, the change in SLD value of the target lesion is compared to determine the presence or absence of non-target lesions and to look for new lesions. Efficacy is then determined: complete remission (CR), partial remission (PR), stable disease (SD), and disease progression (PD).

**Table S1B** A simplified version of the RECIST criteria for categorizing outcomes in evaluating the therapeutic effects of drugs for pulmonary nodules.

| Classification | | Definition |
| --- | --- | --- |
| DCR (Disease Control Rate) | CR (Complete response) | Disappearance of all lesions |
|  | PR (Partial response) | >30% decrease SLD |
|  | SD (Stable disease) | no PR - no PD |
| PD (Progressive disease) | | ≥20% increase SLD* compared to smallest SLD in study  or clinical outcomes of resection |

According to the criteria above, the presence of any new and well-defined malignant tumor lesion indicates disease progression. If the new lesion is ill-defined, e.g. due to small size, further evaluation will clarify the cause. If the evaluation of a definite lesion is repeated, then progression should be documented on the date of the first evaluation. Lesions found in previously unscanned areas were considered new lesions. Since this study was mainly aimed at the evaluation of isolated lung nodules, without considering tumor dissemination and implantation or evaluating lymph node metastasis, the evaluation criteria were simplified by simply measuring the longest diameter of the target lung nodule and taking the results of the two thin-layer CT follow-up images as the evaluation time points. The simplified evaluation criteria are summarized below.

**Table S2** Comparison of Lung Nodule Parameters Between Treatment and Control Groups.

|  | Control | Treatment | P value | OR (95%CI) |
| --- | --- | --- | --- | --- |
| Risk reduction (%) | 62 (55.4) | 66 (70.2) | 0.0286* | 1.901(1.051 to 3.424) |
| Risk increased (%) | 50 (44.6) | 28 (29.8） |  |  |
| PD (%) | 13 (11.61) | 2 (2.13) | 0.0091** | 6.04 (1.501 to 27.35) |
| DCR (%) | 99 (88.39) | 92 (97.87) |  |  |

**Table S3** Details of patients whose samples were used for multiple immunofluorescences.

| Patient | Sex | Age | Stage | Group | Pathological Subtype |
| --- | --- | --- | --- | --- | --- |
| #1 | F | 69 | IA | Treatment | Microinvasive adenocarcinoma |
| #2 | F | 59 | IA | Treatment | Invasive lung adenocarcinoma, grade ll, mainly lepidic type, a small part of acinar type |
| #3 | F | 34 | IA | Control (blank) | Microinvasive adenocarcinoma |
| #4 | M | 67 | IA | Control (Antibiotic) | Invasive lung adenocarcinoma, grade l, acinar type |

**Table S4** Attributable Toxicities. Number of Events (Number of Patients)

| **Toxicity** | **Events** | **Control** | **Treatment** |
| --- | --- | --- | --- |
| Diarrhea | 2 (162) | 0 (90) | 2 (72) |
| Abdominal pain | 0 (162) | 0 (90) | 0 (72) |
| Cough | 0 (162) | 0 (90) | 0 (72) |
| Rash | 0 (162) | 0 (90) | 0 (72) |


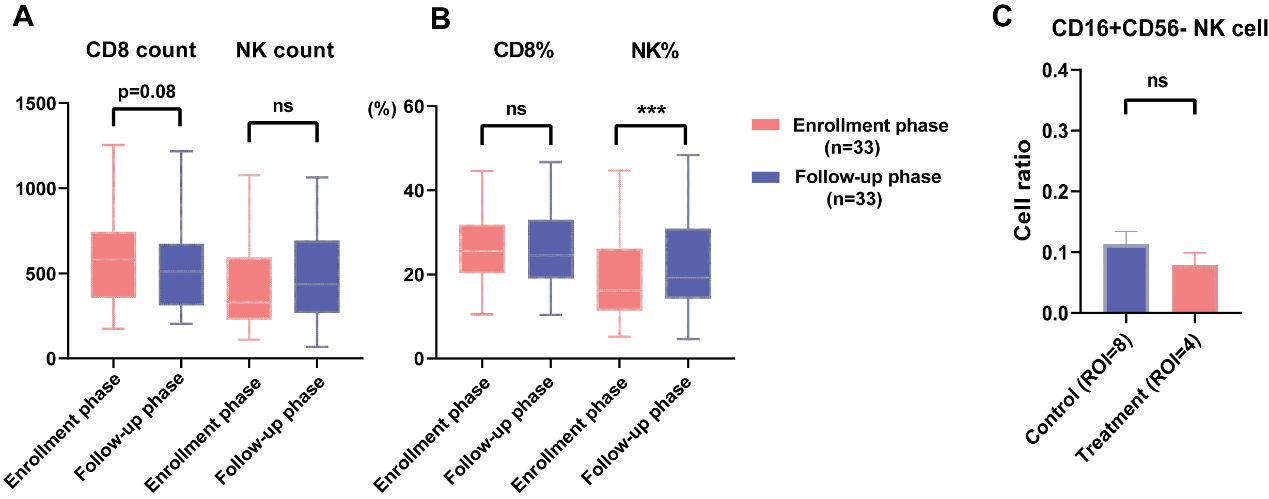
**Figure S1** The bar graph shows the absolute counts (A) and percentages (B) of NK cell subpopulations and CD8 cell subpopulations in the peripheral blood of patients in the treatment group at baseline and follow-up. the Y-axis represents the number or percentage of cells; the X-axis shows the different immune cell subpopulations; and the enrolment stage and follow-up data are indicated by different colors. (C) The histograms show differences in the proportion of CD16+CD56- NK cells in the intra-tumoral regions of pulmonary nodule samples from the control group (ROI=8) and the treatment group (ROI=4). The data represents the quantitative analysis of mIF data that is shown in Figure 4C.
